# Atmospheric Corrosion, Antibacterial Properties, and Toxicity of Silver Nanoparticles Synthesized by Two Different Routes

**DOI:** 10.1155/2020/8891069

**Published:** 2020-12-10

**Authors:** I. DeAlba-Montero, Claudio A. Ruiz-Torres, Diana P. Portales-Pérez, Fidel Martínez-Gutierrez, Félix Echeverría, Martha E. Compeán-Jasso, Yolanda G. Cataño-Cañizales, Facundo Ruiz

**Affiliations:** ^1^Doctorado Institucional en Ingeniería y Ciencia de Materiales, Universidad Autónoma de San Luis Potosí, San Luis Potosí, Mexico; ^2^Facultad de Ciencias, Universidad Autónoma de San Luis Potosí, San Luis Potosí, Mexico; ^3^Department of Chemical and Biomolecular Engineering & KAIST Institute of Nanocentury, Korea Advanced Institute of Science and Technology (KAIST), Daejeon 34141, Republic of Korea; ^4^Facultad de Ciencias Químicas, Universidad Autónoma de San Luis Potosí, San Luis Potosí, Mexico; ^5^Grupo de Corrosión y Protección, Ingeniería de Materiales, Universidad de Antioquia, Medellín, Colombia

## Abstract

Silver nanoparticles (AgNPs) have been widely employed or incorporated into different materials in biological application, due to their antibacterial properties. Therefore, antimicrobial capacity and cytotoxicity have been highly studied. However, most of these reports do not consider the possible corrosion of the nanomaterials during their exposure to atmospheric conditions since AgNPs undergo a transformation when they come in contact with a particular environment. Derived from this, the functionality and properties of the nanoparticles could decrease noticeably. The most common silver corrosion process occurs by the interaction of AgNPs with sulfur species (H_2_S) present in the atmospheric air, forming a corrosion layer of silver sulfide around the AgNPs, thus inhibiting the release of the ions responsible for the antimicrobial activity. In this work, AgNPs were synthesized using two different methods: one of them was based on a plant extract (*Brickellia cavanillesii*), and the other one is the well-known method using sodium borohydride (NaBH_4_). Chemical stability, corrosion, antibacterial activity, and toxic activity were evaluated for both sets of prepared samples, before and after exposition to atmospheric air for three months. The structural characterization of the samples, in terms of crystallinity, chemical composition, and morphology, evidenced the formation of link structures with nanobridges of Ag_2_S for non- “green” AgNPs after the air exposition and the intact preservation of silver core for the “green” sample. The antibacterial activity showed a clear improvement in the antimicrobial properties of silver in relation to the “green” functionalization, particle size control, and size reduction, as well as the preservation of the properties after air exposition by the effective “green” protection. The cytotoxicity effect of the different AgNPs against mononuclear cells showed a notable increment in the cell viability by the “green” functionalization.

## 1. Introduction

The constant development of the medical, industrial, and scientific sectors requires the need for more efficient materials, adaptable to more rigorous conditions without reducing their properties, and remains a profitable solution. In recent years, nanotechnology has represented solutions of high efficiency in the resolution of problems in a wide variety of applications and areas. An ideal case to exemplify this issue is the use of silver nanoparticles, due to their antimicrobial properties and such high surface area has been widely used in a broad range of sectors such as household, personal care, textiles, baby bottles, washing machines, refrigerators, and food containers [1–4]. Every day more and more products use silver nanoparticles. However, this has raised some red flags and raised some important questions to answer. Are these nanoparticles safe for your health and for the environment? Could microorganisms become resistant to the effects of silver? In view of this, recent studies have extensively studied the toxicity of AgNPs [1], where the biological effects of these type of NPs have been analyzed using microorganisms, various cell lines, and nonvertebrate and vertebrate model organisms [2].

Furthermore, it is well known in materials science that in the application of nanomaterials formed of pure metals, chemical stability plays an important role, due to the high reactivity of that kind of materials, as a consequence of their large surface area, they exhibit kinetics of corrosion more accelerated and therefore a high instability to the air exposition. This fact can reduce significantly their performance and lifetime. In relation to this, silver-based nanomaterials show a huge susceptibility to corrosion in response to the strong affinity of silver for chemical species such as sulfide and chloride [5–7], reducing remarkably their reactivity, antimicrobial properties, and applicability; hence, this phenomenon represents a severe problem for the areas and industries in which the use of this type of nanomaterials predominates.

Moreover, it has been reported that silver atmospheric corrosion effects after several weeks of exposure are represented mainly by its interaction with reduced sulfur ligands presents in the surrounding environment, forming the passivation of the particles surface represented by a core-shell composed of a thin layer of silver sulfide (Ag_2_S), which hinders the reaction and interaction of the Ag^0^ core, modifying the consequently their transport, reactivity, and toxicity [3, 4, 8].

In relation to the above, nowadays, functionalization of materials and organometallic science has developed important advances in the stabilization and protection of nanoparticles in order to lengthen the durability of the materials. The protection process occurs through the incorporation of a protective shell that modifies the interactions of the NPs with the surrounding medium and promotes the selective reactivity [9]. The nature of the coating agent or shell could have an inorganic or organic composition, highlighting the organic-metal systems, due to the material properties upgrade and particle size reduction [5–7, 10]. In functionalization or stabilization of materials, the interaction of the organism with the inorganic surface occurs through processes of physical absorption or chemical interactions between the ligand groups contained in the organic molecule and the surface atoms of the NPs. A case to be highlighted is the molecules that contain the ligand group carboxyl owing to the bidentate coordination capacity of the nanomaterials surface by the presence of the ligand groups –OH and =O, producing the efficient grafting of organic brushes and formation of the stabilizer housing [11–13].

Additionally, in reference to nanoparticles synthesis and stabilization, currently, the “green” synthesis methods have been extensively studied owing to their striking advantages over nongreen methods that involve synthetic chemicals. The use of eco-friendly materials such as plant extracts provides several benefits over chemical and physical methods, principally because of comprising cost-effective, environment-friendly, and easily scalable synthesis methodologies, absent of high pressure, energy or temperature conditions, and toxic chemicals. [14].

In fact, the main benefits of “green” synthesis methods in nanoparticles obtaining are directly related to the “green” reducer agent in response to the fact that the natural extract functions similarly as a functionalizing agent in response to the carboxyl ligand group presence in the tannin molecules contained in the bioextracts, producing an organic shell that modifies the interactions of the particles with the surrounding medium and potentiating its properties. In view of this, the use of “green” functionalization agent in AgNPs synthesis could stabilize the particles through size reduction by effects of steric repulsion and furtherance the air stability of the material by the perturbation of Ag surface chemistry affinity. In this work, *Brickellia cavanillesii* was used as bioreducer and biofunctionalizing agent in the synthesis of silver nanoparticles in order to control the particle size, enhance its properties, and grant it anticorrosive stability to atmospheric conditions. To further corroborate the advantages involved in the superficial modification of the particles, AgNPs were obtained by means of “green” and conventional synthesis methodology; “green” (G-AgNPs) and simple (S-AgNPs) nanomaterials were synthesized. The materials were evaluated in terms of their optical properties and crystallinity morphology, and the statistical analysis of the particle size distribution was performed.

Additionally, the stability of the particles in time was analyzed, evaluating their anticorrosive capacity to atmospheric conditions after three months of exposition in the absence and presence of functionalization agent (G-AgNPs_2_ and S-AgNPs_2_). The corrosion present in the particles was characterized by X-ray diffraction (XRD), transmission electron microscopy (TEM), and energy dispersive spectroscopy (EDS). The reactivity of the materials in function of Ag^0^ content consumption was analyzed by the evaluation of the antimicrobial properties and the cytotoxicity of the materials.

## 2. Materials and Methods

### 2.1. Reagents

Leaf extract of *Brickellia cavanillesii* (purchased in a local natural product supermarket) and sodium borohydride (NaBH_4_, Sigma Aldrich, ACS reagent) were used as reducer agents to prepare AgNPs. Silver nitrate (AgNO_3_, Sigma Aldrich, ACS reagent) was used as a silver precursor, and NH_4_OH (30% w/w aqueous solution, Sigma Aldrich, ACS reagent) was used to adjust the pH. Dulbecco's Modified Eagle Medium (DMEM, Gibco™), fetal bovine serum (FBS), penicillin, streptomycin, 3-(4, 5-dimethyl-2-thiazolyl)-2, 5-diphenyl-2H-tetrazolium bromide (MTT), dimethylsulfoxide (DMSO), NaCl, CaCl_2_, phosphate-buffered saline (PBS), and Mueller–Hinton broth were purchased from Sigma Aldrich.

### 2.2. Silver Nanomaterials Synthesis

#### 2.2.1. “Green” Synthesis

To prepare the *Brickellia cavanillesii* extract, 10 g of powdered plant leaves was transferred into a beaker containing 200 mL of freshly boiled distilled water. The obtained extract was filtered with a Whatman filter paper 125 mm and stored at 4°C. A 0.01 M solution of silver nitrate was prepared in an Erlenmeyer flask. Posteriorly, 10 mL of plant extract (bioreducer agent) was added to 100 mL of silver nitrate solution keeping a concentration of 0.01 M. The pH of the reaction was adjusted in the range of 10-11 by the addition of NH_4_OH; the solution was magnetically stirred for 30 min.

#### 2.2.2. Nongreen Synthesis

For nongreen synthesis, aqueous solutions of 0.01 M silver nitrate and 0.02 M sodium borohydride were prepared. 150 ml of sodium borohydride solution was placed into the Erlenmeyer flask. Afterwards, 50 ml of silver nitrate solution was added drop by drop to the NaBH_4_ solution, the Erlenmeyer flask was placed in an ice bath, and a magnetic bar stir was used. A few drops of 1.5 M NaCl solution were added until solution evidence of a color change is noticeable. The final solution was magnetically stirred for 30 min. No other chemical compound was used as stabilizer.

The reduction of Ag^+^ ions to Ag^0^ and the nanoparticles formation was evidenced by the solution color that change from colorless to dark amber. Later on, the nanoparticle solutions were covered to avoid any further reaction and stored properly for future use. Samples were identified as G-AgNPs-1 (green synthesis) and S-AgNPs-1 (nongreen synthesis).

### 2.3. Corrosion of Silver Nanoparticles

The stability of AgNPs synthesized by “green” synthesis and nongreen synthesis against atmospheric corrosion was analyzed by exposing the materials to local atmospheric conditions in an open container for three consecutive months (average temperature 21°C, relative humidity 29%, and concentration of sulfur dioxide in the air (SO2) 3.33 ppm). The samples exposed to the air were identified as G-AgNPs-2 and S-AgNPs-2, respectively. The values of the atmospheric conditions were provided by a certified local monitoring station. It is expected that, in addition to the SO_2_ species, other sulfur species such as SOx and H_2_S were present in the air, but we have no evidence for them. On the other hand, it is possible that the corrosion of silver nanoparticles was observed in less time but it was decided to evaluate them after a period of 3 months.

### 2.4. Physical Characterization Methods

Optical properties of fresh (non-air exposed) and air exposed AgNPs were evaluated through UV-Vis spectroscopy, using a S2000 UV-Vis spectrometer from Ocean Optics Inc. The “green” synthetized AgNPs were analyzed by Fourier Transform Infrared (FTIR) spectroscopy and thermogravimetric analysis (TGA) in order to quantify the presence of the organic compound that coated the NPs. Infrared spectrums were made with an IR Affinity–1 spectrometer from Shimadzu instrument. TGA was performed using a DSC calorimeter (Brand Waters Model DSC 500). Morphological, size measurements, and elemental distribution of S and Ag in the fresh and air-exposed nanoparticles were evaluated by transmission electron microscopy (TEM) and energy dispersive spectroscopy (EDS) performed in a JEOL JEM-1230 at an acceleration voltage of 100 kV. To estimate size average and sizes distribution, the sizes of 300 particles were measured.

Structural modifications induced by the corrosion process were evaluated by X-ray diffraction. XRD patterns were collected using a GBC-Difftech MMA diffractometer with filtered CuK*α* (*λ* = 1.54 A) radiation. To confirm the crystal phase modification of air exposed nanoparticles and quantify the presence of Ag and Ag_2_S, X-ray data were subjected to Rietveld refinement [50] using the program MAUD.

### 2.5. Antimicrobial Test

#### 2.5.1. Bacterial Strains

Four bacteria were evaluated, two Gram-negative *Escherichia coli* (ATCC 25922) and *Pseudomonas aeruginosa* (ATCC 25853) and two Gram-positive *Staphylococcus aureus* (ATCC 29213) and *Enterococcus faecalis* (ATCC 29212).

#### 2.5.2. Antimicrobial Activity of AgNPs

The antibacterial activity was evaluated by the standard microdilution method (CLSI M100-S25 January 2015) [15], which determines the minimum inhibitory concentration (MIC, as the minimum concentration of tested substance that inhibited the growth of the bacterial strain) and the minimum bactericidal concentration (MBC, as the minimum concentration of tested substance that kills the bacterial strain). The MIC was determined on 96-well microdilution plates. Microorganisms (105 CFU/mL) were exposed to serial dilutions of AgNPs with Mueller Hinton Broth (Fluka), and the endpoints were determined when no turbidity in the well was observed after 24 hours of incubation at 37°C. Minimum bactericidal concentration (MBC) was determined by culturing on agar plates from two wells before and two wells after the minimum inhibitory concentration breakpoint, after 24 hours of incubation at 37°C. All assays were carried out in triplicate for all the strains tested.

### 2.6. Cytotoxicity Measurements

#### 2.6.1. Isolation of Mononuclear Cells and Cell Culture

In order to carry out the experimental in vitro study, AgNPs solutions with different concentration were prepared using the silver-based nanomaterials (green and nongreen samples) with and without exposition to corrosion conditions. Suspensions of blood mononuclear cells from 8 healthy young men aged between 25 and 32 years were prepared as well. The entirety voluntary subjects gave a written informed consent prior to their participation in the study. 15 mL of their venous blood was taken, placed in heparinized propylene plastic tubes, and shaken for 10 min. Then, carefully a layer of 35 mL of diluted cell suspension over 15 mL of Ficoll-Hypaque (96%) in a 50 mL conical tube was added and centrifuged at 2500 rpm for 20 minutes, so, the mononuclear cells (lymphocytes, monocytes, and thrombocytes) were collected and washed with PBS and transferred to a new 50 mL conical tube to centrifuge at 1500 rpm for 15 minutes. Subsequently, the supernatant was removed carefully and transferred to the conical tube with PBS, centrifuged at 1300 rpm for 15 minutes, and similarly the supernatant was completely removed. Posteriorly, the cells were resuspended in supplemented DMEM with penicillin (100 g/ml), streptomycin (100 g/ml), and 10% FBS.

#### 2.6.2. MTT Assay

Once mononuclear cells were isolated, the viability of the cells after exposure to the silver nanomaterials was evaluated by the amount of viable cells stained by 3-(4, 5-dimethylthiazol-2-yl)-2, 5-diphenyltetrazolium bromide, which was released using dimethylsulfoxide (DMSO, Sigma Aldrich) MTT (3-(4, 5-dimethylthiazol-2-yl)-2, 5-diphenyltetrazolium bromide) assay that constitutes a measure of energy generating a potential of the cell and assess the mitochondrial function by measuring electron transfer potential [16]. The mononuclear cells were plated in 96-well plates and exposed to both types of AgNPs synthesized (G-AgNPs and S-AgNPs). Cells were transferred into at concentrations of 25, 50, 100, 200, and 300 mg/L (ppm) and maintained in a humidified atmosphere at 37°C and 5% CO2. After 4, 24, and 48 h, the medium was removed from each well and replaced with new medium containing MTT solution in an amount equal to 10% of culture volume and incubated for 4 h at 37°C until a purple colored formazan product developed. The resulting formazan product was dissolved in DMSO, and the absorbance was measured at 570–690 nm by using a Synergy HTX Multo-Mode Microplate Reader (BioTek Instrument Inc.).

## 3. Results and Discussion

### 3.1. “Green” Synthesis of AgNPs by *Brickellia cavanillesii* as Reducing Agent

**Figure 1:**
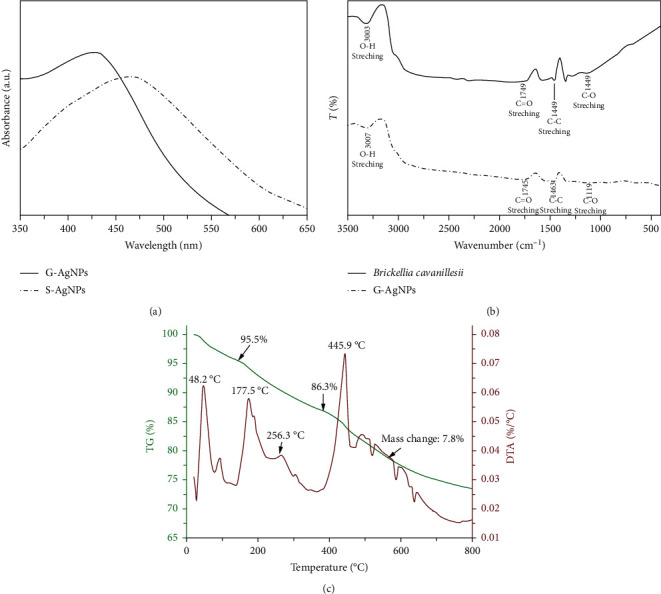
(a) UV-Vis absorption spectra of G-AgNPs and S-AgNPs, respectively. (b) FTIR spectrum of *Brickellia cavanillesii* and G-AgNPs. (c) Thermogravimetric analysis of fresh AgNPs by “green” synthesis.

In the G-AgNPs “green” synthesis method reported in this work using leaves extract of *Brickellia cavanillesii*, the reduction of Ag^+^ to Ag^0^ occurred by the action of polyphenols, mainly tannins groups, by the extract acting as a bioreducer agents for the silver ions, in response to the pH change; the –OH groups contained in the tannins suffered a hydrolysis releasing a hydrogen atoms and electrons that subsequently reduce the Ag^+^ ions, initiating the process of formation of primary particles. Posteriorly, the particles functionalization occurs simultaneously to the nucleation process by the bidentate coordination of the ligand group carboxyl with the Ag-atoms contained in the NPs surface, preventing the interaction, agglomeration, and growth of the particles. The biosynthesis process was evidenced by the solution color change to brown, indicating the formation of silver nanoparticles; the “green” synthesis of NPs was monitored by UV-Vis spectroscopy, observing the characteristic band of Ag at 429 nm after synthesis conclusion (Figure 1(a)). The increase in color of the solutions is directly proportional to the reaction time, in response to the excitation of the surface plasmon resonance effect (SPR) and the reduction of AgNO_3_ [17]. Similarly, after the completion of the synthesis of S-AgNPs, the particles exhibited the characteristic excitation peak of Ag plasmon resonance at 465 nm (Figure 1(a)). The blue shift and narrowing of the G-AgNPs band compared to the second described sample is related to the effects of quantum confinement in relation to the presence of smaller particles [1].

#### 3.1.1. “Green” Silver Nanoparticles Functionalization

The nanoparticles obtained by “green” synthesis methodology were characterized by FTIR spectroscopy and TGA analysis in order to corroborate the biofunctionalization of the particles induced for the plant extract.

*(1) FTIR*. The FTIR spectra of *Brickellia cavanillesii* extract present four principal peaks associated to the deformation vibrational modes of the principal functional groups of the material, located at 3003 cm^−1^ (stretching of O-H), 1749 cm^−1^ (stretching of aromatic C=O), 1449 cm^−1^ (stretching of aromatic C-C), and 1148 cm^−1^ (stretching of aromatic C-O); additionally, the functional groups associated with the vibrational frequencies shown coincide with the functional groups that make up the tannin molecule. The vibrational frequencies characteristic of *Brickellia cavanillesii* extract are in a similar way presented by G-AgNPs with a slight shift to 307, 1745, 1463, and 1119, respectively, corroborating the functionalization of AgNPs by the biofunctionalizing agent.

The presence of organic on silver NPs surface as the coated agent is related to the coordinate chemistries interaction among the carboxyl ligand groups contained in tannins molecules with Ag-atoms to conform metal-ligand bonds. Indeed, the phenomena involved in NPs functionalization can be described by crystalline field theory, explaining the interaction of lone pair electron available on the ligand group with the empty d-orbitals of Ag-atoms contained on particle surface through its lodging and developing the formation of coordinate covalent bonds and generating the particles functionalization [10, 18, 19] (Figure 1(b)).

*(2) TGA analysis*. The TGA analysis was performed in order to corroborate the G-AgNPs functionalization by tannins. The results are shown in Figure 1(c), which displays the mass loss dependence of the samples expressed as a percentage of the initial mass and temperature. Two distinct mass loss peaks can be seen in the results, a weak peak centered at 48.2°C, indicating weight loss of almost 1.77% related to the phenomena of photopolymerization, thermal reforming, preliminary oxidation steps, and elimination of volatile fractions. At 256.3°C, is found a second sharper peak associated with the beginning of tannins degradation represented by a loss weight of 5.14% and it could be the result of the partial breakdown of the intermolecular bonding.

**Figure 2:**
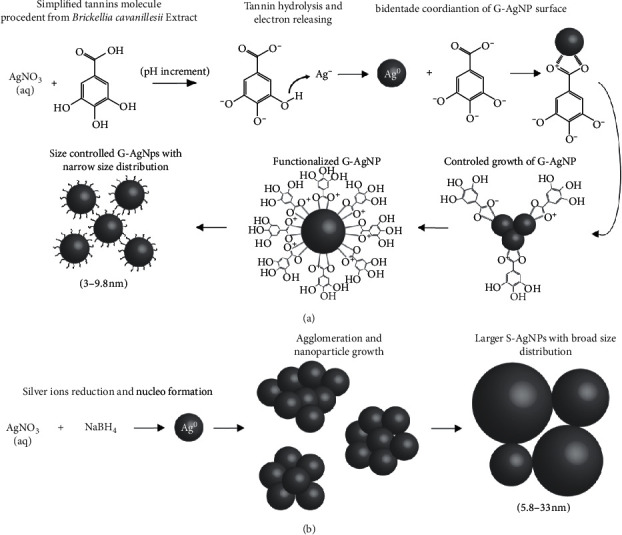
Schematic illustration showing: (a) synthesis and functionalization mechanism of G-AgNPs by tannins (simplified molecule) contained in *Brickellia cavanillesii* extract and particle size-control; (b) synthesis of S-AgNPs using NaBH4 and uncontrolled growth of the particles.

Finally, the third degradation of organic groups takes place evidenced by a remarked peak at 445.9°C [20] (Figure 1(c)). In view of this information, it could be established that the functionalization of silver NPs occurs by tannins acting as “green” coated agent. Furthermore, through the “green” functionalization presence on the particles, it forms a protective layer that modifies the properties and interactions of the material with the surrounding medium, increasing its stability uncontrolled particle growth and to control their corrosion by reducing the reactivity with sulfur species present in the environmental (Figure 2).

### 3.2. Structural Characterization

The “green” and nongreen NPs were structurally characterized to further evaluate the effects of the “green” functionalization agent in the structure of the particles.

**Figure 3:**
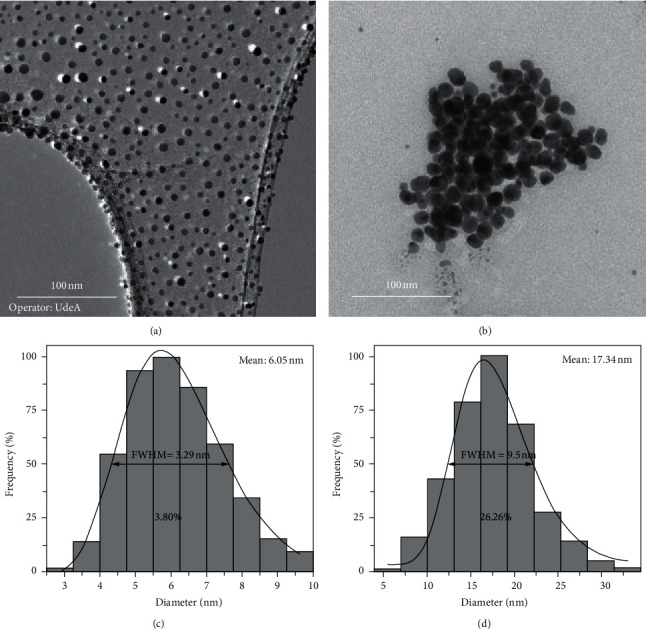
TEM micrograph of silver nanoparticles. (a) AgNPs obtained by “green” synthesis. (b) AgNPs obtained by the nongreen method. Differential size distribution of (c) G-AgNPs and (d) S-AgNPs.

The morphological analysis correspondingly to G-AgNPs illustrated in the TEM images present particles with a spherical shape (Figure 3(a)). The particles are disposed in a monodisperse manner; this behavior could be directly related to the functionalization of the particles produced by the “green” synthesis method, inducing steric repulsion effects among the nanoparticles by osmotic pressure presence or volume exclusion between the coated agent's organic brushes grafted in the particles surface, stabilizing them and arrangement the G-AgNPs well-dispersed. Nonetheless, respectively to S-AgNPs, the particles exhibit more irregular morphologies, presenting pseudo-spherical shapes (Figure 3(b)); moreover, the disposition contrasts severely with the sample described above, displaying the agglomeration of the particles and its arrangement in nanoclusters, in response to the lack of coated agent, producing the approaching and agglomeration of the NPs.

#### 3.2.1. Size Distribution Analysis

Statistical analysis of the size distribution of the different AgNPs synthesized was carried out to further contrast the size-control and predominance of small diameters of particles in relation to the presence of a “green” functionalizing agent. Over 300 particle diameters were measured using TEM photomicrographs of the samples. The statistical parameters obtained are presented in Table 1. The differential size distribution of Ag particles displays a histogram with a size scattered in the range of 3.06–9.78 nm presenting an asymmetric geometry positively biased and an average particle size of 6.05 nm. The calculated coefficient of variation CV (%) presents a value of 3.8% indicating quite a narrow distribution and control in the size (Figure 3(c)). On the other hand, the sample S-AgNPs presents such different behavior in the particle size and size distribution. The diameters range is in the range of 5.8–33.10 nm, which represent in a first instance a notable increment in size. The histogram presents a partial symmetric geometry and a calculated average size of 17.34 nm. The CV (%) obtained for this material is 26.26%, showing quite an increase in size and a broad size distribution in contrast to the sample described above (Figure 3(d)). The notable difference in the size control between AgNPs samples is directly related to the presence of the “green” functionalizing agent that surrounds the particles. In relation to this fact, during the nanoparticle nucleation process, the particles surface modification occurs by grafting organic brushes produces steric repulsion effects among the particles avoiding the agglomeration and growth of these, controlling the particles size and the size distribution. In view of that, the remarkable increment in size and the broad size distribution correspondingly to S-AgNPs is caused by the vulnerability of the particles to get closer to each other, agglomerates, and grows to bigger particles.

The cumulative distribution for AgNPs by “green” synthesis method presents a scattered of the diameters where only 10% have sizes higher than 7.15 nm, indicating that the 90% of the particles are smaller than this size, corroborating the size-control of the G-AgNPs attributed to the “green” synthesis methodology. Furthermore, for the case of S-AgNPs, 90% of the particles sizes have diameters lower than 22.04, where only 10% are smaller than 9.76 nm, predominating the large sizes and broad size distribution (Table 1).

### 3.3. Chemical Stability Corrosion Resistance of “Green” Silver NPs

Freshly prepared silver nanoparticles (G-AgNPs-1 and S-AgNPs-1) and particles exposed to atmospheric conditions (G-AgNPs-2 and S-AgNPs-2) were evaluated by TEM and EDS in order to get evidence of the corrosion of the nanoparticles surface and the possible formation of the of Ag_2_S shell over the silver core. Furthermore, the antimicrobial capacity and cytotoxicity of the materials were also evaluated with the purpose of analyzing the effect of the NPs corrosion in the antibacterial properties and the cytotoxicity in the presence and absence of “green” organic coated.

#### 3.3.1. Structural Characterization: XRD

**Figure 4:**
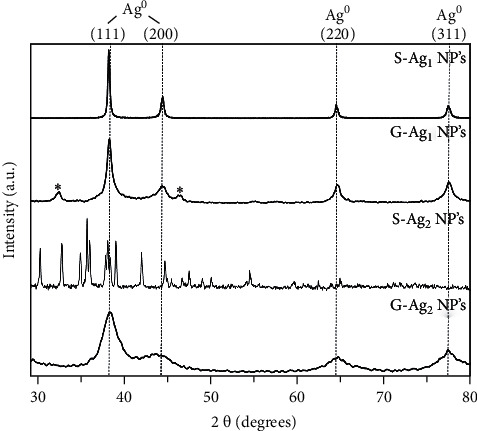
X-ray diffraction (XRD) patterns of “green” and nongreen silver NPs before and after the sulphuration process.

XRD diffractograms were acquired to further analyze the crystallinity and the corrosion grade of the silver nanoparticles. Additionally, the sulfurized silver crystal phase on the samples was evaluated by XRD data Rietveld refinement. The diffractogram related to S-AgNPs_1_ shows the peaks associated to the Ag cubic phase at 38.10 44.20, 64.45, and 77.39°, which can be indexed as (111), (200), (220), and (311) planes (JCPDS File No. 04–0783) (Figure 4). By using Rietveld refinement, a crystallite size of 39.1 nm (Table 2) was calculated. Furthermore, the XRD data associated with the sample G-AgNPs-1 display the peaks correspondingly to Ag located at similar positions and additionally show associated a crystallite size value of 10.0 nm (Figure 4; Table 2). Moreover, it is possible to observe a remarkable widening and decrease of the intensity of the peaks in contrast with the first sample described, these effects are directly associated with the particle size reduction of G-AgNPs due to is decreasing the number of atoms available to form the crystallites, inducing several defects on crystals developing a partial amorphous behavior that produce the widening of the diffracted peaks [21].

The XRD analysis of the samples corresponding to the corrosion process (S-AgNPs-2) nonexposed the lack of the Ag crystal phase evidencing an advanced sulphuration (Figure 4). Rietveld refinement of the XRD data confirmed the presence of 100% of silver sulfide in the sample, conforming of *α*-Ag_2_S (acanthite; Table 2), which is the only thermodynamically stable crystal phase of Ag_2_S; hence, comparing this information with previous studies [6, 11], it can be argued that the corrosion process of the particles occurs firstly by the consumption of the Ag^0^ nucleus and the formation of a core-shell structure of Ag/−Ag_2_S, finally, the sulphuration gives as a result that Ag_2_S becomes the main phase present. It is possible that a very small nucleus of the metallic Ag remains, but the used techniques are not able to detect it. As a consequence of the corrosion process, a noticeable modification of the material properties is expected. Therefore, it can be observed a difference in response to this phenomenon for biocoated and non-biocoated nanoparticles by observing their functionalities.

In addition, the crystal size related to the conventional silver sample was calculated with a value of 44.1 nm (Table 2), representing a crystal increment of 11.3%, produced possibly to the recrystallization of the cubic phase Ag^0^ to the monoclinic phase *α*-Ag_2_S, which is a more complex structure with different stoichiometry and physical-chemical properties, producing the increment in the crystal dimension.

On the other hand, the sample G-AgNPs-2 showed a completely stability against corrosive conditions. The spectra display the characteristic peaks of Ag^0^ (Figure 4) and the data refinement shows the existence of 100% of this crystalline phase in the sample (Table 2). The anticorrosive properties against atmospheric conditions are attributed to the “green” functionalization. The biocapping agent composed of tannins acts as a protective layer and prevents the particles corrosion by the interaction with sulfurized chemical species present in the environment. In addition, the exhibited peaks by G-AgNPs_2_ in XRD results present a minimal widening compared with that G-AgNPs_1_, and, similarly, the calculated crystal size value of 6.60 nm shows a slight decrease, which could be possibly related to nonsignificant corrosion effects on nanoparticles surface, slightly modifying the crystal net.

#### 3.3.2. Morphological Characterization: TEM and EDS

**Figure 5:**
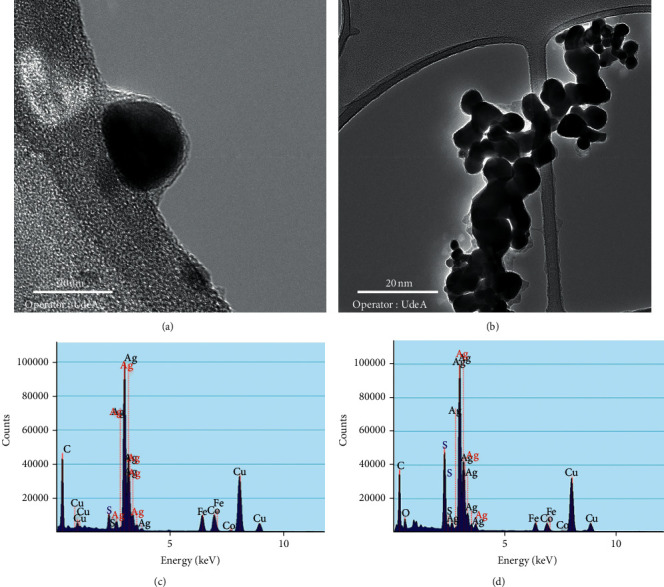
TEM photomicrography of (a) G-AgNPs-2 and (b) S-AgNPs-2. Energy dispersive spectroscopy (EDS) of silver nanoparticles (c) G-AgNPs and (d) S-AgNPs.

The morphological characterization of materials after corrosion process was carried out in order to identify the presence of sulphuration structures on the samples. The TEM image corresponding to the sample G-AgNPs-2 shows a similar structure in comparison with the original sample previous the corrosion test, demonstrating the preservation of the silver core and the effectiveness of the biofunctionalization agent to avoid the sulphuration of the particles (Figure 5(a)).

In the case of S-AgNPs-2, it is possible to observe quite a different behavior, showing the complete modification of the morphology and the presence of corrosion structures (Figure 5(b)). The presence of corrosion structures is associated with the interaction of the silver contained in the different particles with the sulfur species present in the air, forming chains of *α*-Ag_2_S composition, chains that bind the particles and arrange them in bridge-type structures [6, 11].

Energy dispersive spectroscopy (EDS) was used to analyze silver nanoparticles elemental composition and evaluate the sulfur present in the samples exposed to air. The elemental analysis corresponding to G-AgNPs-2 samples presents such a high predominance of Ag with 91.48% and a minor concentration of S with a value of 8.52 (Figure 5(c); Table 3), corroborating the corrosion resistance of the “green” samples. The detection of the small presence of sulfur in the sample in contrast to the XRD analysis may be due to the fact that the EDS analysis evaluates the elemental composition at one point in the sample, in contrast to the diffracted beams that analyze the sample entirety. In addition, despite the slight corrosion of particles surface in response to minor discontinuities in the protective layer of the particles, this could represent the formation of a core-shell structure, composed of a slightly corroded silver shell surrounding the Ag^0^ core. As matter of fact, it has broadly reported how, in metallic structures, the formation of core-shell compounds from the sulfured phase of the metal can prevent further oxidation of the remaining nucleus [22]. Therefore, the presence of a thin layer of acanthite in G-AgNPs_2_ and the “green” functionalization can act synergistically give a greater anticorrosive stability to the particles [10].

**Figure 6:**
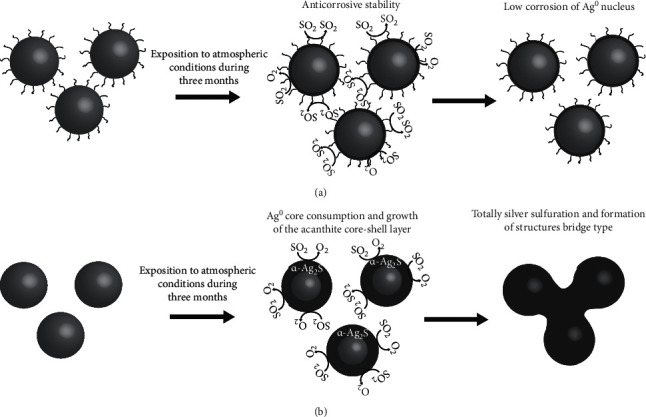
Schematic illustration showing (a) anticorrosive stability against sulfurs species present in the air of G-AgNPs and (b) sulfuration and instability of S-AgNPs to atmospheric conditions.

The EDS analysis of the sample S-AgNPs_2_ exhibits an elemental proportion of 61.98 and 38 for Ag and S, respectively (Figure 5(d); Table 3), coinciding with the stoichiometric ratios of the crystalline compound *α*-Ag_2_S. In view of the information described in this section and information previously reported, the possible corrosion mechanism of the different silver samples synthesized in this work is established as illustrated in the schematic model shown in Figure 6.

#### 3.3.3. Effect of Corrosion on Antimicrobial Activity

Primarily, as reference model, the fresh silver materials were tested to further measure their antibacterial capacity; MIC values were obtained for both AgNPs (“green” and non-green synthetized), tested against *E. coli* (ATCC 25922), *S. aureus* (ATCC 29213), *E. faecalis* (ATCC 29212), and *Pseudomonas aeruginosa* (ATCC 25853). The results are presented in Table 4. AgNPs obtained by “green” synthesis presents such superior antibacterial activity, up to four times more than S-AgNPs-1 against the four strains tested (Table 4). The remarkably difference among G-AgNPs-1 and S-AgNPs-1 could be related to, firstly, the quite high size control and the narrow size distribution of G-AgNPs-1 nanoparticles, because a greater uniformity in the sizes of nanoparticles induces a better functioning and reactivity of these [10]. Secondly, the increase colloidal stability in response to the materials functionalization promotes better nanoparticles reactivity against the target, which is evidenced by the uniform arrangement of the well-dispersed NPs due to the green functionalization. Therefore, it is expected that the functionalized materials showed a greater antibacterial action in compare with S-AgNPs-1. Finally, previous reports demonstrate the size influence of silver nanoparticles in the antimicrobial mechanism for particles with diameters less than 10 nm. NPs with size smaller than 10 nm can easily penetrate into the bacteria's cellular membrane, increasing their bactericidal properties, and additionally, the bacteria have lower resistance to this type of particles [17].

On the other hand, in particles with diameters >10 nm, the bactericidal dominant mechanisms are the ions realizing from silver cores and the interaction of them with the bacteria [23]. As a result, the modification of the properties of the “green” nanomaterial and the fact that >90% of the particles have sizes smaller than 7.65 nm and explain the remarkedly difference between the antimicrobial activities of the silver different NPs. Additionally, the results correspondingly to AgNPs synthesized by nongreen method are similar to that reported by Martínez-Castañón et al. [24]. In general, the MIC associated with the Ag materials present low values on the tests against *E. coli*, *S. aureus,* and *P. aeruginosa* unlike the essays against *E. faecalis*. These results could be related to differences between the cell wall of each strain; the cell wall of *E. faecalis* is a Gram-positive which is wider than the cell wall of Gram-negative strains.

**Figure 7:**
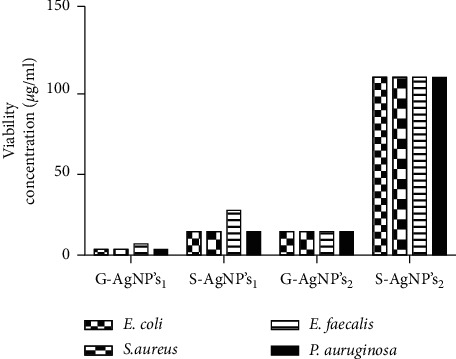
Comparison of bactericidal activity of Ag nanomaterials before and after atmospheric exposure (corrosion process).

Posteriorly, the nanoparticles were exposed to the atmospheric conditions for 3 months; the MIC and MBC of the materials were similarly evaluated, obtaining quite different results in comparison with the non-air-exposed NPs (Table 4). The different in the antimicrobial activities are related to silver corrosion, due to the particles surface passivation through the strong interaction of sulfurous chemical species with the Ag^0^, forming a layer of Ag_2_S. The corrosion layer presence in the NPS drastically modifies the dispersibility and colloidal stability of the particles, reducing the activity and reactivity. In addition, surface passivation makes the silver ionization and the interaction with the bacteria difficult, diminishing the antibacterial activity, as a consequence of the entire transformation of Ag^0^ to the crystal phase acanthite and inactivating the particles as agent antimicrobial. On the other hand, the G-AgNPs-2 sample shows a slight increase in antibacterial activity, increasing their MIC to 13.75 ± 0.0 for all strains. Demonstrating the benefits of biofunctionalization in AgNPs air corrosion resistance for antimicrobial properties preservation, Figure 7 illustrates the antibacterial activity of the different silver nanomaterials before and after environmental exposure.

#### 3.3.4. Effect of AgNPs Corrosion in the Cytotoxicity

**Figure 8:**
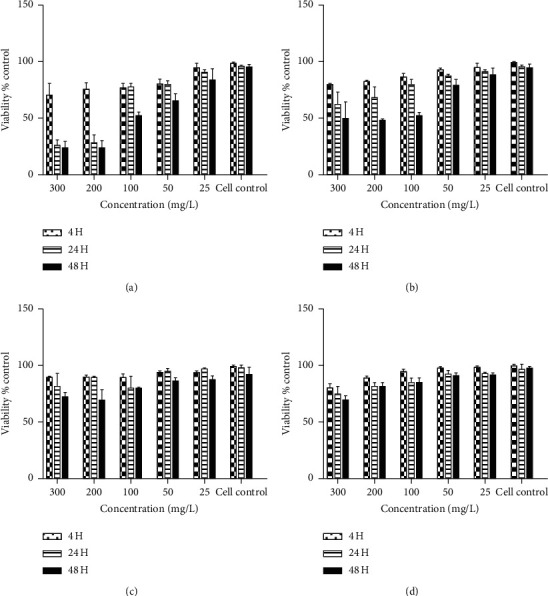
MTT assay to test the effect of AgNPs on the viability of mononuclear cells (MNC). The MNC cells were treated with AgNPs at a concentration of 25, 50, 100, 200, and 300 mg/L for 4, 24, and 48 h. At the end of the incubation period, mitochondrial function was determined by the MTT reduction assay as described in Materials and Methods. The MNC treated with (a) S-AgNPs-1, (b) G-AgNPs-1, (c) S-AgNPs_2_, and (d) G-AgNPs-2. The values represent the mean standard deviation of three experiments; to compare the mean values between the control and treatment groups, Student's *t*-test was applied (*p* < 0.05 for each).

The cytotoxicity of the different silver NPs samples nonexposed and exposed to environment was evaluated using the MTT assay, in order to analyze the “green” functionalization effect on cell viability and the modification of the cytotoxic properties as a result of the silver sulphuration. The cytotoxicity of both non-air-exposed samples (G-AgNPs-1 and S-AgNPs-1) showed a decreasing in the cell viability of mononuclear cells in a dose-dependent way. The S-AgNPs-1 samples presented a cytotoxic effect at a dose of 200 mg/L from the first four hours after adding sample addition, leading to a cell viability of 73%, which markedly decreases to 24% and 21% after 24 and 48 h of exposition, respectively. Demonstrating the high cytotoxicity for nanomaterial (Figure 8(a)), on the contrary, the “green” non-air-exposed sample presented cytotoxicity values quite different for similar concentration dose, displaying a cell viability of 80% at 4 h of cell exposition. Posteriorly to 48 h of exposition, the cell viability decreased to 49%, contrasting with the S-AgNPs-1 sample results at the same conditions. However, even after the addition of the maximum NPs dose concentration (300 mg/L), similar values of cell viability persist (Figure 8(b)). Therefore, the “green” synthesis method and the nanomaterial functionalization reduce the silver cytotoxicity. The modification of the cytotoxic properties by the “green” functionalization of the nanoparticles is produced by the polyphenols (tannins) present in nanoparticles surface, preventing the interaction of silver with essential amino acid contained in the cells, such as the cysteine, which contains thiol groups, highly vulnerable to interact with silver, reducing the cytotoxicity of the material, and incrementing the cell viability [30].

Regarding the cytotoxic properties of the air-exposed samples, the S-AgNPs-2 sample displayed cell viabilities values quite different in comparison with the non-air-exposed sample. Showing a high cell viability even at the maximum concentration of NPs after 48 hrs of exposition (Figure 8(c)), which is associated with the total sulphuration of the Ag^0^ nucleus, the rapid precipitation of the particles due to the insolubility of Ag_2_S and the lack of ionizable Ag are ionized to develop a cytotoxic effect. Finally, the case associated with G-AgNPs-2 presents a slightly increment and reduction in cell viability in comparison with G-AgNPs-1 and S-AgNPs-2, respectively (Figure 8(d)). Associated with the slight sulphuration and passivation of NPs surface are reducing the silver ion liberation and the toxic effect of AgNPs, in the same way as in the antimicrobial test section.

## 4. Conclusions

In this work, it was demonstrated the production of resistance-corrosion size-controlled silver nanoparticles by a “green” synthesis methodology, employing *Brickellia cavanillesii* as bioreducer and biofunctionalizing agent for the potentiation of the silver properties in terms of stability against corrosion in atmospheric conditions, the increase and preservation of the antibacterial capacity, and the reduction of the cytotoxicity of the nanomaterial. The synthesis and functionalization occur simultaneously due to the presence of polyphenols (tannins) in the “green” extract, which modify the surface of the particles through coordinated covalent bonds between the ligand groups (−OH) and the Ag atoms, producing stabilization of the particles, obtaining a narrow size distribution (3.8%), and providing them with corrosion resistance properties. The materials were compared with nanometric silver obtained by nongreen methodology in absence of stabilizing agent. The samples were exposed for three months to atmospheric conditions. The evaluation of the morphology and structure of the “green” samples by TEM, EDS, and XRD does not show significant corrosion effects after the air exposition, in contrast to the sample without biocoating, which showed a total transformation of the crystalline Ag^0^ to the *α*-Ag2S crystalline phase after the air exposure. The antimicrobial capacity of the “green” samples against Gram-positive and Gram-negative bacteria showed a remarkable superiority and a slight diminution even after three months of exposure to atmospheric conditions. Through biofunctionalization, it was possible to reduce the cytotoxicity of the material, in response to the decrease in the interaction between the silver and cells in function of the functionalization.

## Figures and Tables

**Table 1:** Size distribution statistical parameters of silver nanoparticles in nm.

Sample	Mean	CV (%)	FWHM	D10	D50	D90
G-AgNPs	6.05	3.8	3.29	4.04	5.67	7.65
S-AgNPs	17.34	26.26	9.5	9.76	15.71	22.04

**Table 2:** Rietveld refinement of the silver nanomaterials before and after corrosion process.

Sampleº	Ag^0^ % in weight	Crystallite size (nm)	*α*-Ag_2_S % in weight	Crystallite size (nm)	Error (%)
S-AgNPs-1	100	33.9	0	ND	±9.32
G-AgNPs-1	100	8.4	0	ND	±1.67
S-AgNPs-2	0	ND	100	44.1	±0.32
G-AgNPs-2	100	6.6	0	ND	±3.90

ND, no data.

**Table 3:** Elemental composition of EDS analysis showed in Figures 5(c) and 5(d) (wt.%).

Sample	Ag (%)	S (%)
G-AgNPs-2	91.48	8.52
S-AgNPs-2	61.98	38

**Table 4:** Minimum inhibitory concentrations of Ag nanomaterials.

MIC of silver nanoparticles (mg/ml)				
Sample	Bacterial strains			
	*E. coli*	*S. aureus*	*E. faecalis*	*P. aeruginosa*
	(ATCC 25922)	(ATCC 29213)	(ATCC 29212)	(ATCC 25853)
G-AgNPs-1	3.37 ± 0.0	3.37 ± 0.0	6.68 ± 0.0	3.37 ± 0.0
S-AgNPs-1	13.75 ± 0.0	13.75 ± 0.0	26.75 ± 0.0	13.75 ± 0.0
G-AgNPs-2	13.75 ± 0.0	13.75 ± 0.0	13.75 ± 0.0	13.75 ± 0.0
S-AgNPs-2	107 ± 0.0	107 ± 0.0	107 ± 0.0	107 ± 0.0
Amikacin	1 ± 0.0	2 ± 0.0	128 ± 0.0	4 ± 0.0
*Brickellia cavanillesii*	−a	−a	−a	−a
Tannic acid	−a	−a	−a	−a

−a, no antibacterial activity was found with concentrations used in this work.

## Data Availability

Data are available on request to the corresponding author.
